# Effect of inspiratory muscle-loaded exercise training on peak oxygen uptake and ventilatory response during incremental exercise under normoxia and hypoxia

**DOI:** 10.1186/s13102-020-00172-1

**Published:** 2020-04-15

**Authors:** Takeshi Ogawa, Maiko Nagao, Naoto Fujii, Takeshi Nishiyasu

**Affiliations:** 1 Division of Art, Music, and Physical Education, Osaka Kyoiku University, Kashiwara, Osaka, Japan; 2 Faculty of Health and Sport Sciences in University of Tsukuba, Tsukuba, Japan

**Keywords:** Hypoxia, Inspiratory muscle training, Ventilation, VO2peak

## Abstract

**Background:**

Although numerous studies have reported the effect of inspiratory muscle training for improving exercise performance, the outcome of whether exercise performance is improved by inspiratory muscle training is controversial. Therefore, this study investigated the influence of inspiratory muscle-loaded exercise training (IMLET) on peak oxygen uptake (*V*O_2peak_), respiratory responses, and exercise performance under normoxic (N) and hypoxic (H) exercise conditions. We hypothesised that IMLET enhances respiratory muscle strength and improves respiratory response, thereby improving *V*O_2peak_ and work capacity under H condition.

**Methods:**

Sixteen university track runners (13 men and 3 women) were randomly assigned to the IMLET (*n* = 8) or exercise training (ET) group (*n* = 8). All subjects underwent 4 weeks of 20-min 60% *V*O_2peak_ cycling exercise training, thrice per week. IMLET loaded 50% of maximal inspiratory pressure during exercise. At pre- and post-training periods, subjects performed exhaustive incremental cycling under normoxic (N; 20.9 ± 0%) and hypoxic (H; 15.0 ± 0.1%) conditions.

**Results:**

Although maximal inspiratory pressure (PImax) significantly increased after training in both groups, the extent of PImax increase was significantly higher in the IMLET group (from 102 ± 20 to 145 ± 26 cmH_2_O in IMLET; from 111 ± 23 to 127 ± 23 cmH_2_O in ET; *P* < 0.05). In both groups, *V*O_2peak_ and maximal work load (W_max_) similarly increased both under N and H conditions after training (*P* < 0.05). Further, the extent of W_max_ decrease under H condition was lower in the IMLET group at post-training test than at pre-training (from − 14.7 ± 2.2% to − 12.5 ± 1.7%; *P* < 0.05). Maximal minute ventilation in both N and H conditions increased after training than in the pre-training period.

**Conclusions:**

Our IMLET enhanced the respiratory muscle strength, and the decrease in work capacity under hypoxia was reduced regardless of the increase in *V*O_2peak_.

## Background

There are some difficulties in attaining success in hypoxic training for improving sea-level competitive performance among athletes; this is related to the lack of sufficient absolute training intensity caused by a substantial decrease in aerobic capacity [1]. In the acute hypoxic (H) condition, peak oxygen consumption (*V*O_2peak_) is reduced by impaired gas exchange due to a reduction in the ambient partial O_2_ pressure. The effect of hypoxia (2400–3000 m) on *V*O_2peak_ [2, 3] is more pronounced in subjects with higher *V*O_2peak_ with large decrease in haemoglobin O_2_ saturation (SaO_2_). Notably, individuals with a large increase in maximal minute ventilation (*V*_Emax_) under acute H condition, relative to those under normoxic (N) condition, show a smaller reduction in *V*O_2peak_ [4, 5], suggesting that a high *V*_E_ under H condition is beneficial for minimising the reduction in *V*O_2peak_. However, high *V*_E_ under H condition leads to higher O_2_ cost in the respiratory muscles than that under N condition [6]. Since respiratory muscle work during heavy exercise accounts for 10–15% of the whole body *V*O_2_ [7] even under N condition, a compromised blood flow to the active muscle during heavy exercise was observed [8, 9]. Further, diaphragm fatigue has been observed following strenuous exercise [10, 11], which limits exercise performance [12, 13]. In this regard, under H condition, an increase in *V*_E_ with a minimum increase in respiratory muscle work may be essential to prevent a greater reduction in the *V*O_2peak_ and exercise performance under H condition.

Inspiratory muscle training (IMT) has confirmed the improvement of respiratory muscle strength as a consequence of an increased cross-sectional area [14] in the diaphragm, thereby reducing respiratory muscle fatigue and dyspnoea after an exhaustive exercise [15, 16]. Some studies reported that IMT improved exercise performance without any change in *V*O_2peak_ [17, 18]. However, numerous studies have reported the effect of IMT by attempting to improve exercise performance [19–24], and the outcome of whether exercise performance is improved by IMT is controversial. The discrepancy in the outcomes of IMT would be influenced by study design, training protocol, subject fitness level, and type of exercise test. With regard to training protocol, since IMT is often conducted at a resting condition as well as during exercise, pulmonary ventilation is not high. Indeed, isocapnic hyperventilatory training was reported to improve exercise performance [25, 26]. Thus, it is postulated that applying inspiratory load during exercise with hyperventilation could have more effect on improving *V*O_2peak_ and work capacity. In fact, McEntire et al. [27] reported that 15% PImax inspiratory load-added exercise training greatly improves inspiratory muscle strength and exercise tolerance compared with exercise training alone.

With regard to the effect of IMT on hypoxic exercise, Esposito et al. [28] reported that 8 weeks of IMT did not enhance *V*O_2peak_ and work capacity under hypoxia (11% O_2_). Further, Downey et al. [15] reported that exercise tolerance of 85% *V*O_2peak_ constant treadmill running did not change after 4 weeks of IMT. Since the importance of ventilatory response is more emphasised in gas exchange under H condition than under N condition, it remains a possibility that inspiratory muscle-loaded exercise training (IMLET) can improve *V*O_2peak_ and work capacity to a greater extent in H condition than in N condition. Therefore, this study aimed to investigate the influence of IMLET on *V*O_2peak_, respiratory responses, and exercise performance under N and H exercise conditions. We hypothesised that IMLET enhances respiratory muscle strength and improves respiratory response and therefor *V*O_2peak_ and work capacity under H condition.

## Methods

### Subjects

Sixteen healthy young volunteers who belong to the university track club (13 men and three women) were recruited for this study. Ten were middle and long-distance runners, and six were sprinters. All subjects did not have any cardiac, pulmonary, and musculoskeletal disease. Subjects were asked to maintain their current diet and regular physical training during the study period. All subjects were instructed to hold similar daily training. Before each testing day, subjects were instructed to avoid vigorous exercise and intake of alcohol and caffeine within 24 h. They were randomly divided into the IMLET group (7 men and 1 woman; age, 19.6 ± 0.9 years; height; 1.71 ± 0.09 m; body mass, 57.4 ± 4.9 kg) and exercise training (ET) alone group (6 men and 2 women; age, 19.5 ± 1.4 years; height; 1.68 ± 0.06 m; body mass, 54.5 ± 4.9 kg). The influence of the menstrual cycle could not be excluded in women; thus, pre- and post-exercise tests were performed at the follicular stage.

This study was approved by the Human Subjects Committees of the Osaka Kyoiku University in accordance with the guidelines set forth by the Declaration of Helsinki. All subjects provided verbal and written informed consent before participating in this study.

### Experimental design

**Fig. 1:**
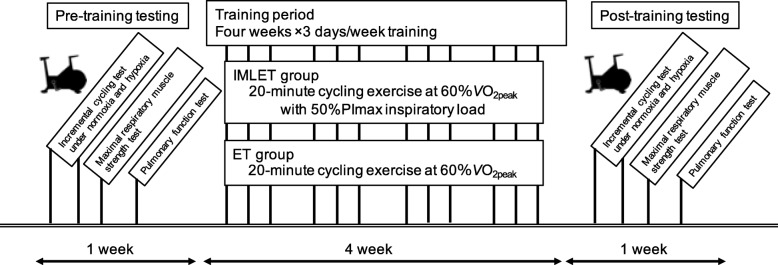
Experimental design. Participants performed the incremental cycling tests under normoxia and hypoxia (15%O_2_), maximal respiratory muscle strength test, and pulmonary function test within 1 week in the pre- and post-training period. Participants (*n* = 16) were randomly divided into the inspiratory muscle-loaded exercise training (IMLET, *n* = 8) and exercise training (ET, n = 8) groups. Exercise training on a cycle ergometer at 60% of VO_2peak_ with 50% of PImax inspiratory muscle-loaded during exercise (IMLET) or at 60% of VO_2peak_ (ET) for 20 min/day, 3 days/week, for 4 weeks

Figure 1 presents the experimental design. Initially, subjects were instructed to familiarise themselves to the test protocols and training protocol. As pre-training period tests, all subjects performed the pulmonary function test and respiratory strength test under N condition. Incremental cycling tests (see below for details) were performed under N (20.9 ± 0.0%) and H (15.0 ± 0.1%) conditions for 1 week. Incremental cycling tests were separately performed in random order with at least 48-h intervals. After the baseline test, all subjects completed 4 weeks of training. Exercise training was conducted 3 days per week for a total of 12 sessions. Since subjects conducted athletic training five times per week, IMLET and ET sessions were held at three times per week. After the training period, subjects performed the same tests as those at baseline (post-training tests).

### Exhaustive incremental exercise test

All subjects performed incremental cycling tests until exhaustion in our laboratory (placed at 132 m above sea level) under N and H conditions. The room temperature was 20.2 ± 0.4 °C, and the room was continuously ventilated to minimise increases in CO_2_ concentration in the air. Under H condition, hypoxic gas was generated by adding moistened N_2_ gas to ambient air from packed large Douglas bags (700 L). Under both conditions, inspiratory gas was supplied through a pipe from large Douglas bags (700 L). Subjects breathed through a mouthpiece attached to a pneumotachograph (MLT1000L; AD-Instrument, Sidney, Australia) and a two-way valve (2700; Hands Rudolph, Inc., KS, USA). Subjects started to breathe air 3 min before starting recording of measurements. The incremental cycling test was performed on a cycle ergometer (E-828; Monark, Vansbro, Sweden). Before beginning the exercise, subjects were kept at rest to obtain resting values; subsequently, subjects performed 5 min of warm-up cycling at 30 watts. The pedalling rate was set at 60 rpm. The initial pedalling load was set at 60 watts. The pedalling load was subsequently increased by 15 watts every 1 min until exhaustion. The achievement of exhaustion was assumed when the pedalling rate was below 55 rpm despite strong verbal encouragement. Metabolic and ventilatory variables were calculated using the metabolic cart (AE-310 s; MINATO Medical; Osaka, Japan). The variables were measured breath-by-breath and calculated at 60 s. All subjects accomplished two of the following three criteria for *V*O_2peak_: *V*O_2_ did not increase further despite increases in work load (increase in < 2.0 ml kg^− 1^ min^− 1^) (achieved by 63% of the subjects), the respiratory quotient was > 1.1 (achieved by 93% of the subjects), or the maximal heart rate was > 90% of the age-predicted value (achieved by 93% of the subjects). Moreover, all subjects rated 20 on the Borg scale and were exhausted despite strong encouragement.

To estimate the work of breathing (WOB), oesophageal and mouth pressures were measured using a pressure transducer catheter (MicroSensor Basic Kit: Codman & Shurtleff, Inc., MA, USA). Airflow was measured using a pneumotachograph (MLT1000L; AD-Instrument; Sidney, Australia). The variables were recorded at a sampling rate of 200 Hz using Power Lab (Power Lab 8/35; AD-Instrument, Sydney, Australia) and analysis software (LabChart 7; AD-Instrument, Sydney, Australia). Before and after the test measurements, a pressure transducer catheter was carefully calibrated. To obtain calibration signal, a pressure transducer catheter was immersed to 0–60 cm depth in a darken pipe with water. A pneumotachograph was calibrated by using a 3 L calibration syringe. An oesophageal pressure catheter was inserted from the nasal passage to a distance 1/4 of the height minus 9 cm through a nasal cavity and further inserted to the stomach where the oesophageal pressure was confirmed to be changing from negative to positive. Thereafter, the catheter was carefully withdrawn to keep the negative pressure at rest condition (within − 1 to − 10 cmH_2_O). The mouth pressure catheter was fixed to the mouthpiece. Trans-pulmonary pressure was calculated as a difference between the oesophageal pressure and mouth pressure. Since the participants often failed to have inspiratory capacity manoeuvre, lung volume was not estimated during exercise. Thus, the lung volume was reset at the end of the expiration flow. The estimated WOB was calculated as an integration of trans-pulmonary pressure and volume curve. We assessed the tidal volume (VT), respiratory frequency (fR), V_E_, trans-pulmonary pressure, peak expiratory flow rate (PEFR), and WOB every 60 s.

Heart rate was measured using the three-lead electrocardiogram (FE132: AD-Instrument; Sydney, Australia). SaO_2_ was measured using a forehead pulse oximeter (N-560; Covidien; CA, USA). Maximal achieved work load (W_max_) and time to exhaustion were measured to assess the exercise performance.

### Pulmonary function and respiratory muscle strength tests

Pulmonary function and respiratory muscle strength were assessed by spirometry (AS-507, MINATO Medical, Osaka, Japan) according to manufacturer instruction. The subjects familiarised the test protocol before the test day to avoid the learning effect. The vital capacity (VC), forced vital capacity (FVC), and forced expiratory volume in 1 s (FEV_1.0_) were measured in at least three trials. The subjects wore a nose clip and performed the test while standing. The highest value of 3–4 trials was taken as the value for each parameter. The pulmonary function assessment also included the maximal voluntary ventilation performed at 12 s of maximal ventilatory effort. Respiratory muscle strength was assessed using a handheld mouth pressure meter (AS-507, MINATO Medical, Osaka, Japan).

The respiratory muscle strength was assessed as the static maximal inspiratory and expiratory mouth pressure. All tests were performed in the standing position. Similarly, the subjects familiarised the test protocol before each testing day to avoid the learning effect. The inspiratory muscle strength was evaluated by maximal inspiratory mouth pressure (PImax) measurement. Initially, subjects expired slowly until residual volume (RV) and then performed maximal inspiratory effort from RV. The expiratory muscle strength was evaluated by the maximal expiratory mouth pressure (PEmax) measurement. Initially, subjects inspired slowly until the total inspiratory capacity (IC) and then performed maximal expiratory effort from IC. Both PImax and PEmax values were calculated as a mean value of 1 s including the highest value when maximal pressure was held at least 1.5 s. The values were assigned as the best of at least five satisfactory efforts. Subjects were given verbal encouragement for performing the maximal effort.

### Training protocol

The subjects were assigned to the IMLET group or ET group. At the training day, the IMLET group performed 20 min of cycling exercise (60 rpm) at 60% *V*O_2peak_ of each subject calculated from incremental exercise test under normoxia. The training intensity was determined as the percentage of the intensity of *V*O_2peak_ observed. Through the exercise, inspiratory pressure was loaded at 50% PImax by using inspiratory muscle trainer (POWERbreatheK5; IMT Technologies, Birmingham, UK). POWERbreatheK5 requires a predetermined inspiratory pressure throughout the inspiration for opening the electrically controlled shutter, while expiration is not resisted. The subjects were not given any instruction about breathing depth, frequency, or volume during IMLET exercise. Previous studies have reported that a load of the 50% PImax of IMT elicits an adaptation to the respiratory muscles [20, 22]. Inspiratory load was calculated from the pre-training period muscle strength test of PImax, and this load was used throughout the IMLET. Meanwhile, the ET group performed 20 min of cycling exercise (60 rpm) at 60% *V*O_2peak_ without any resistive inspiratory pressure.

### Statistical analysis

Data are expressed as mean ± standard deviation. SPSS 25 (IBM, Armonk, NY, USA) was used for all statistical analyses. Variables obtained by respiratory function test were analysed with a specific mixed-model analysis of variance (ANOVA) with training period variables (pre vs. post-training) and subject groups (IMLET vs. ET). Variables obtained during the incremental exercise test were analysed with three-way repeated-measures ANOVA with factors of the experimental conditions (H vs. N), training period, and subject groups. The comparisons of the extent of change by conditions or training period were analysed with two-way repeated-measures ANOVA with the training period variables and subject groups. After detecting the main effect, post-hoc Bonferroni multiple comparisons were performed. Paired t-tests were used to compare the variables of pairwise comparison. The effect size (Cohen’s *d* (*d*) in the pairwise test and squared partial eta (*η*_*p*_^*2*^) in ANOVA) was calculated when a significant difference was found. *P* values < 0.05 were considered statistically significant.

## Results

Participant characteristics are shown in Table 1. No differences in physical and fitness at the pre-training measurement variables were found between the groups. Moreover, no significant differences in age, height, weight, PImax, and *V*O_2peak_ at pre-test were observed between the IMLET and ET groups.

**Table 1:** Variables of the characteristics of subjects

	IMLET (*n* = 8)	ET (*n* = 8)	*P* value	Effect size (d)
Sex [M / W]	7/1	6/2		
Age [years]	19.6 ± 0.9	19.5 ± 1.4	0.82	0.11
Height [cm]	171.3 ± 8.9	167.5 ± 5.5	0.31	0.59
Weight [kg]	57.4 ± 4.9	54.5 ± 4.9	0.3	0.50
PImax [cmH_2_O]	101 ± 15	104 ± 22	0.64	0.33
*V*O_2peak_ [ml kg^− 1^ min^− 1^]	55.1 ± 7.2	53.4 ± 7.7	0.45	0.10

Values are mean ± SD. PImax: maximal inspiratory pressure, *V*O_2peak_ Peak oxygen consumption

### Respiratory function and respiratory muscle strength

The results of the pulmonary function test and respiratory muscle strength test are shown in Table 2. Only VC significantly increased by 4.7 ± 4.2% after post-training vs at pre-training in the IMLET group (*P* < 0.05, *d* = 0.48). Further, FVC significantly increased in both training groups (2.2% in IMLET and 2.5% in ET).

**Table 2:** Variables of pulmonary function test and respiratory muscle strength test

	IMLET (*n* = 8)		ET (*n* = 8)		ANOVA		
	pre	post	pre	Post	period	group	interaction
Vital capacity [L]	4.1 ± 0.4	4.3 ± 0.5*	4.2 ± 0.7	4.2 ± 0.7	*P* = 0.14, ηp^2^ = 0.15	*P* = 0.94, ηp^2^ = 0.00	*P* = 0.02, ηp^2^ = 0.34
Force vital capacity [L]	4.1 ± 0.4	4.2 ± 0.5	4.0 ± 0.6	4.1 ± 0.6	*P* = 0.02, ηp^2^ = 0.34	*P* = 0.81, ηp^2^ = 0.00	*P* = 0.29, ηp^2^ = 0.01
Force expiratory volume in one second [L]	3.7 ± 0.4	3.7 ± 0.3	3.7 ± 0.5	3.7 ± 0.5	*P* = 0.78, ηp^2^ = 0.06	*P* = 0.76, ηp^2^ = 0.01	*P* = 0.76, ηp^2^ = 0.01
Maximal voluntary ventilation by 12 s [L min^− 1^]	143 ± 31	148 ± 33	120 ± 35	134 ± 26	*P* = 0.07, ηp^2^ = 0.22	*P* = 0.24, ηp^2^ = 0.09	*P* = 0.35, ηp^2^ = 0.06
PImax [cmH_2_O]	102 ± 20	145 ± 26*	111 ± 23	127 ± 23*	P = 0.01, ηp^2^ = 0.54	*P* = 0.51, ηp^2^ = 0.03	*P* = 0.47, ηp2 = 0.04
PEmax [cmH_2_O]	140 ± 29	169 ± 29*	135 ± 25	155 ± 25*	*P* = 0.00, ηp^2^ = 0.73	*P* = 0.58, ηp^2^ = 0.02	*P* = 0.04, ηp2 = 0.26

**Fig. 2:**
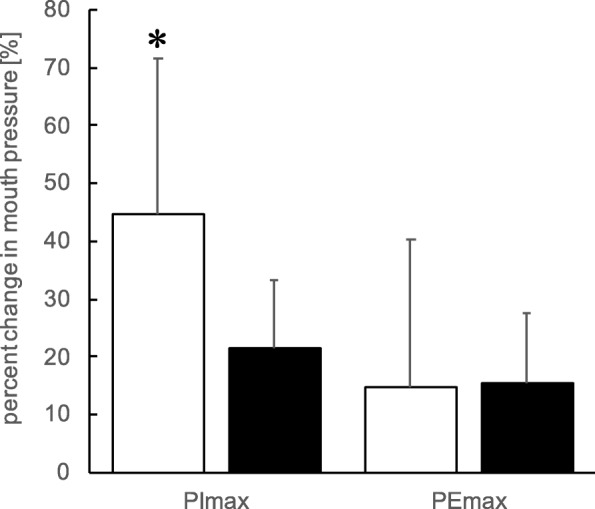
Percentage of change in respiratory muscle strength after the training period. PImax, maximal inspiratory pressure; PEmax, maximal expiratory pressure; white bar represents the inspiratory muscle-loaded exercise training (IMLET) group; black-bar represents the exercise training (ET) group; * *P* < 0.05, IMLET vs. ET. Values are mean ± SD

Values are mean ± SD. *: significantly different from pre-training, *PImax* Maximal inspiratory pressure, *PEmax* Maximal expiratory pressure,

The results of the PImax and PEmax are shown in Fig. 2 and Table 2. PImax significantly increased after post-training vs pre-training in both the IMLET group (44.6 ± 27.3%; *P* < 0.05, *d* = 1.96) and ET group (14.9 ± 11.8%; *P* < 0.05, *d* = 0.68) (Table 2 and Fig. 2). The extent of increase in PImax following training was significantly greater in the IMLET group than in the ET group (*P* < 0.05, *d* = 1.41, Fig. 2). PEmax significantly increased at post-training vs pre-training in both the IMLET group (21.6 ± 25.4%; *P* < 0.05, *d* = 0.82) and the ET group (15.6 ± 12.1%; *P* < 0.05, *d* = 0.81 (Table 2 and Fig. 2). No difference in the extent of increase of PEmax was found between the IMLET and ET groups.

### Incremental exercise test under normoxia

**Fig. 3:**
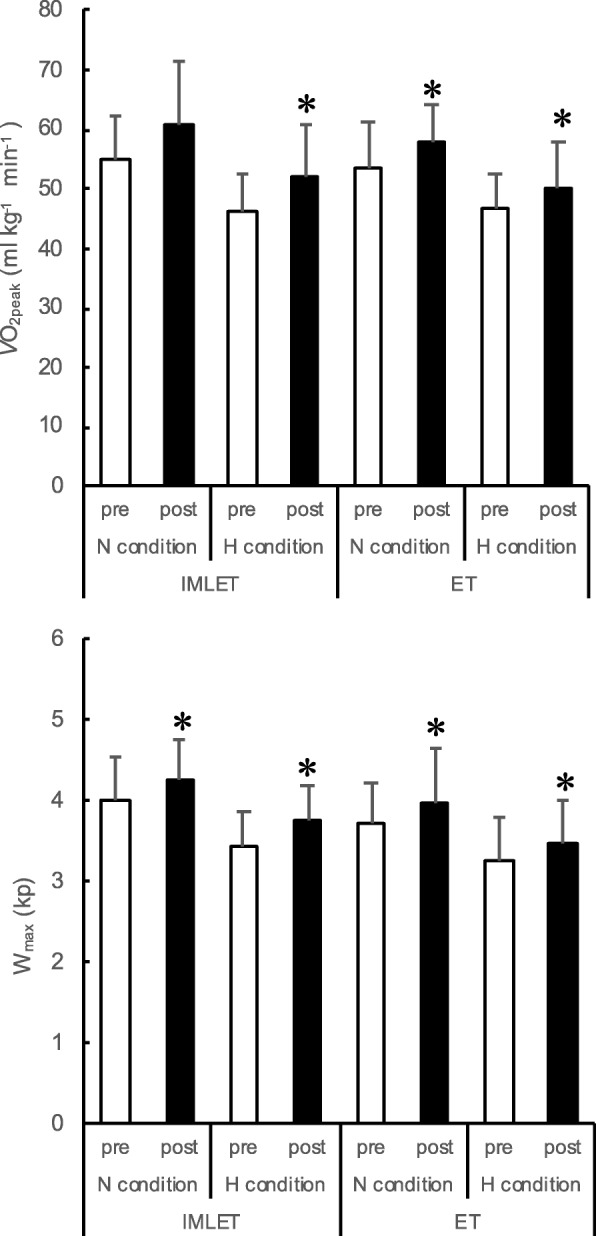
*V*O_2peak_ and W_max_ before and after the training period. *V*O_2peak_, peak oxygen uptake; W_max_, maximal exercise intensity in the incremental exercise test; pre, pre-training period test; post, post-training period test; N, normoxia; H, hypoxia; IMLET, inspiratory muscle-loaded exercise training group; ET, exercise training group;* *P* < 0.05 vs. pre-training test. Values are mean ± SD

Table 3 and Fig. 3 show the variables of the exhaustive incremental test under N condition. At post-training, *V*O_2peak_/w under N condition increased by 9.2 ± 7.8% in the ET group compared with that at pre-training (*P* < 0.05, *d* = 0,67, Fig. 3), whereas in the IMLET group, it increased by 11.0 ± 13.5%, but failed to reach significance (*P* = 0.058, *d* = 0.97, Fig. 3). The percentage of improvement of *V*O_2peak_/w by the training was not significantly different between the training groups. W_max_ (6.4 ± 3.7% in the IMLET; 6.4 ± 7.0% in ET) significantly increased at post-training compared with those at pre-training (Table 3 and Fig. 3). *V*_Emax_ significantly increased by 16.7 ± 9.7% in the IMLET group at post-training vs pre-training *(P* < 0.05, *d* = 0.82). Further, the extent of increase in *V*_Emax_ at post-training vs pre-training was significantly greater in the IMLET group than in the ET group (*P* < 0.05). No significant difference in WOB was found between before and after training in both training groups. Both PIpeak and PEpeak were not changed by the training in both training groups, while PEFR significantly increased by 20.5 ± 23.4% after the training in the IMLET group (*P* < 0.05, *d* = 0.76).

**Table 3:** Maximal values during incremental test under normoxia and hypoxia

	Normoxic condition				Hypoxic condition				ANOVA			
	IMLET (*n* = 8)		ET (*n* = 8)		IMLET (*n* = 8)		ET (*n* = 8)					
	pre	Post	pre	post	pre	post	pre	post	condition	period	group	Interaction
*V*O_2peak_ [L min^− 1^]	3159 ± 464	3487 ± 635	2897 ± 498	3171 ± 489	2631 ± 433	2980 ± 569	2546 ± 424	2717 ± 489	*P* = 0.00, η_p_^2^ = 0.90	P = 0.00, η_p_^2^ = 0.55	*P* = 0.34, η_p_^2^ = 0.07	*P* = 0.35, η_p_^2^ = 0.06
*V*CO_2peak_ [L min^− 1^]	3422 ± 524	3752 ± 584	3298 ± 528	3360 ± 511	2924 ± 383	3356 ± 616	2914 ± 479^#^	3045 ± 511	*P* = 0.00, η_p_^2^ = 0.82	P = 0.00, η_p_^2^ = 0.62	*P* = 0.42, η_p_^2^ = 0.05	*P* = 0.82, η_p_^2^ = 0.00
W_max_ [kp]	4.03 ± 0.51	4.28 ± 0.49	3.72 ± 0.53	3.97 ± 0.54	3.44 ± 0.44	3.75 ± 0.44	3.28 ± 0.53	3.47 ± 0.54	P = 0.00, η_p_^2^ = 0.80	P = 0.00, η_p_^2^ = 0.75	*P* = 0.32, η_p_^2^ = 0.07	*P* = 0.23, η_p_^2^ = 0.10
*V*_Emax_ [L min^− 1^]	109.6 ± 20.6	127.6 ± 23.1	106.2 ± 16.7	109.9 ± 19.7	104.8 ± 22.0	121.8 ± 25.0	103.6 ± 16.4	107.5 ± 19.7	*P* = 0.08, η_p_^2^ = 0.19	P = 0.00, η_p_^2^ = 0.70	P = 0.00, η_p_^2^ = 0.97	P = 0.05, η_p_^2^ = 0.83
*f*_R_ [breath min^− 1^]	50.9 ± 9.7	53.7 ± 11.7	49.2 ± 8.5	48.3 ± 9.3	48.9 ± 12.1	50.3 ± 11.8	50.4 ± 8.6	47.6 ± 9.3	*P* = 0.27, ηp^2^ = 0.08	*P* = 0.91, η_p_^2^ = 0.00	*P* = 0.66, η_p_^2^ = 0.01	*P* = 0.89, η_p_^2^ = 0.01
VT [L]	2.47 ± 0.33	2.81 ± 0.45	2.30 ± 0.38	2.42 ± 0.41	2.46 ± 0.24	2.85 ± 0.44	2.27 ± 0.43	2.66 ± 0.41	*P* = 0.50, η_p_^2^ = 0.03	*P* = 0.01, η_p_^2^ = 0.63	*P* = 0.51, η_p_^2^ = 0.03	*P* = 0.58, η_p_^2^ = 0.02
VE VO_2_^− 1^	34.58 ± 2.6	36.63 ± 2.86	37.08 ± 5.33	34.69 ± 5.04	39.61 ± 3.06	40.83 ± 3.80	40.89 ± 3.58	39.80 ± 5.04	*P* = 0.01, η_p_^2^ = 0.80	*P* = 0.01, η_p_^2^ = 0.93	*P* = 0.89, η_p_^2^ = 0.01	*P* = 0.42, η_p_^2^ = 0.05
VE VCO_2_^− 1^	31.9 ± 2.9	33.9 ± 2.2	32.4 ± 3.3	32.8 ± 3.4	35.6 ± 3.7	36.2 ± 2.7	35.7 ± 2.9	35.3 ± 3.4	*P* = 0.00, η_p_^2^ = 0.72	*P* = 0.15, η_p_^2^ = 0.14	*P* = 0.79, η_p_^2^ = 0.01	*P* = 0.72, η_p_^2^ = 0.01
P_ET_CO_2_ [mmHg]	40.9 ± 2.2	37.2 ± 2.3	40.0 ± 3.7	38.7 ± 3.1	36.6 ± 3.0	34.0 ± 2.1	36.4 ± 2.8	35.9 ± 2.9	*P* = 0.00, η_p_^2^ = 0.81	*P* = 0.03, η_p_^2^ = 0.48	*P* = 0.77, η_p_^2^ = 0.01	*P* = 0.68, η_p_^2^ = 0.01
WOB [J min^− 1^]	276 ± 87	318 ± 98	258 ± 66	288 ± 97	235 ± 59	276 ± 85	231 ± 64	299 ± 97	*P* = 0.80, η_p_^2^ = 0.01	*P* = 0.04, η_p_^2^ = 0.28	P = 0.32, η_p_^2^ = 0.08	*P* = 0.53, η_p_^2^ = 0.03
PEpeak [cmH_2_O]	24.5 ± 11.2	23.2 ± 7.0	26.8 ± 9.6	26.4 ± 11.2	22.1 ± 6.3^#^	22.3 ± 12.6	21.6 ± 5.3	21.5 ± 11.2	*P* = 0.01, η_p_^2^ = 0.43	*P* = 0.61, η_p_^2^ = 0.02	*P* = 0.93, η_p_^2^ = 0.01	*P* = 0.81, η_p_^2^ = 0.01
PIpeak [cmH_2_O]	−27.9 ± 22.8	−37.9 ± 9.7	−35.6 ± 5.3	−38.2 ± 10.0	−33.8 ± 4.6	−32.7 ± 8.7	−34.0 ± 7.3	−39.1 ± 8.3	*P* = 0.78, η_p_^2^ = 0.01	*P* = 0.06, η_p_^2^ = 0.25	*P* = 0.48, η_p_^2^ = 0.04	*P* = 0.11, η_p_^2^ = 0.19
PEFR [L sec^− 1^]	6.39 ± 1.56	7.54 ± 1.46	6.66 ± 1.65	6.63 ± 1.39	5.81 ± 1.29	7.00 ± 1.53	5.35 ± 0.51	6.54 ± 1.39	*P* = 0.02, η_p_^2^ = 0.34	*P* = 0.02, η_p_^2^ = 0.36	*P* = 0.49, η_p_^2^ = 0.04	*P* = 0.13, η_p_^2^ = 0.17
HR_max_ [beat min^− 1^]	185.5 ± 7	187.7 ± 7.5	184.3 ± 10.4	184.5 ± 14.4	175.1 ± 9.4	178.0 ± 8.0	180.4 ± 11.7	181.0 ± 14.4	*P* = 0.00, η_p_^2^ = 0.61	*P* = 0.32, η_p_^2^ = 0.61	*P* = 0.65, η_p_^2^ = 0.02	*P* = 0.66, η_p_^2^ = 0.02
SaO_2_ [%]	96 ± 3	96 ± 2	96 ± 2	97 ± 2	82 ± 3^#^	82 ± 4^#^	83 ± 2^#^	84 ± 2^#^	*P* = 0.00, η_p_^2^ = 0.97	*P* = 0.43, η_p_^2^ = 0.04	*P* = 0.53, η_p_^2^ = 0.03	*P* = 0.01, η_p_^2^ = 0.91

Values are mean ± SD. ^#^, significantly different from normoxic exercise; *VO*_*2peak*_ Maximal oxygen consumption; *V*CO_*2peak*_ Maximal carbon dioxide elimination; *Wmax* Maximal work load; *V*_*Emax*_ Maximal minute ventilation; *f*_*R*_ Respiratory frequency; *VT* Tidal volume; *P*_*ET*_*CO*_*2*_ End-tidal partial pressure of CO_2_; *WOB* Work of breathing; *PEpeak* peak expiratory pressure; *PIpeak* Peak inspiratory pressure; *PEFR* Peak expiratory flow rate; *HR*_*max*_ Maximal heart rate; *SaO*_*2*_ arterial oxyhaemoglobin saturation

### Incremental exercise test under hypoxia

**Fig. 4:**
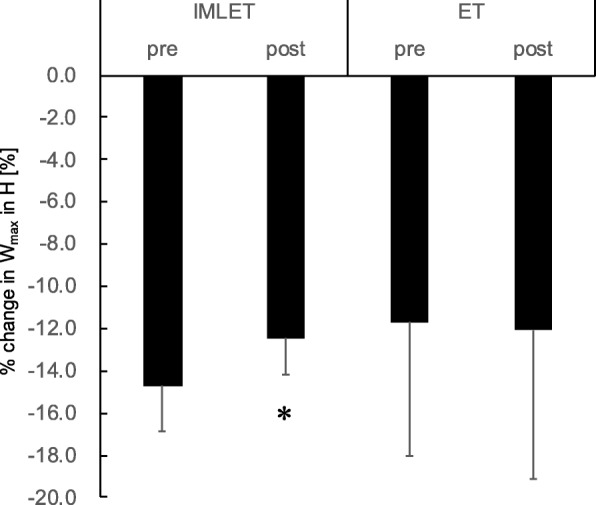
Extent of decrease in W_max_ under H condition (%dW_max_) before and after the training period. W_max_, maximal exercise intensity in the incremental exercise test; pre, pre-training period test; post, post-training period test; H, hypoxia; IMLET, inspiratory muscle-loaded exercise training group; ET, exercise training group;* *P* < 0.05 vs. pre-training test. Values are mean ± SD

Table 3 and Fig. 3 show the variables of the exhaustive incremental test under H condition. *V*O_2peak_/w and W_max_ were significantly lower in the H condition than in the N condition, both at pre- and post-test in all subjects (Fig. 3). Especially, the percentage of decrease in W_max_ under H condition (%dW_max_) at pre- and post-training tests for the IMLET groups was smaller at post-training than that at pre-training (− 14.7 ± 2.2% at pre vs. -12.5 ± 1.7% at post, Fig. 4) (*P* < 0.05, *d* = 1.14). All subjects of the IMLET group showed reduced %dW_max_ at post-training compared with that at pre-training. In the ET group, six of eight subjects showed a greater %dW_max_ at post-training than at pre-training (− 11.7 ± 6.4% in pre-test vs. -12.1 ± 7.0% in post-test). *V*O_2peak_ under H condition increased after the training period in both groups (12.9 ± 13.8% in the IMLET group, 7.4 ± 8.8% in the ET group). The extent of improvement of *V*O_2peak_ by the training was not significantly different between the training groups. Both maximal W_max_ (9.3 ± 3.6% in IMLET, 5.9 ± 5.2%; *P* < 0.05) and time to exhaustion (6.2 ± 4.0% in IMLET, 39. ± 3.4% in CONT; *P* < 0.05) under H condition significantly increased after training compared with those at baseline. *V*_Emax_ under H condition was significantly higher only in the IMLET group at post-training than at pre-training (+ 16.4 ± 8.2%; *P* < 0.05, *d* = 0.72). WOB was increased by 21.9 ± 21.1% at post-training than at pre-training in the ET groups (*P* = 0.05, *d* = 0.84). The extent of increase in WOB in the IMLET group was 22.5 ± 39.5%, but there was no significance (*P* = 0.18, *d* = 0.71). Both PIpeak and PEpeak values were not changed by the training in both training groups, while PEFR significantly increased by 23.9 ± 31.2% at post-training in the IMLET group (*P* < 0.05, *d* = 1.07).

## Discussion

To the best of our knowledge, this study is the first to assess the effect of IMLET on ventilatory response and *V*O_2peak_ under hypoxia. We hypothesised that IMLET would improve the respiratory response and WOB under H condition, resulting in improved gas exchange. Thus, we also hypothesised that IMLET could improve *V*O_2peak_ and work capacity under H condition. First, although both IMLET and ET enhanced the inspiratory and expiratory muscle strength (Table 2), we found that the magnitude of improvement in inspiratory muscle strength was greater in the IMLET group than in the ET group (Fig. 2). Second, under both N and H conditions, *V*O_2peak_ and W_max_ increased in both training groups (Fig. 3); however, *V*_Emax_ increased only in the IMLET group. For exercise under H condition, although similar changes in W_max_ in both training groups were seen, the magnitude of decrease in W_max_ under H condition was significantly smaller in the IMLET groups (Fig. 4). Our results suggest that IMLET changes the ventilatory response under H condition and suppress the extent of decrease in W_max_ under H condition.

### Respiratory muscle strength

Previous studies have reported that 4–10 weeks of respiratory muscle training enhances PImax by approximately 20–30% [15, 19–21]. Our IMLET caused a 44% increase in PImax, suggesting that the respiratory muscle strength improved within a short period. McEntire et al. [27] observed an increase in PImax by 28% following moderate-intensity exercise training with 15% PImax inspiratory loads, although PImax also significantly increased by 6 weeks of 30-min cycling exercise training at 70% peak work rate alone. Compared with that of McEntire et al. [27], we observed an approximately 3-time greater increase in PImax by 50% PImax inspiratory-loaded exercise training. Both McEntire et al. [27] and the present study observed an improvement of PImax in both the IMLET group and ET group. Some previous studies observed increasing respiratory muscle strength caused by strenuous exercise training itself [29–31]. However, we found greater increases in PImax in the IMLET groups than in the ET group, implying that more than 50% PImax inspiratory load needs to highlight the additional influence of inspiratory load on respiratory muscle strength. Interestingly, PEmax also improved after training in both the IMLET and ET groups. Thus, it is speculated that the increase in PEmax after the training period test is caused by the exercise training per se. More likely, although we did not add expiratory load on the exercise training, the diameter becomes smaller because the subject’s mouth is squeezed to hold the inspiratory muscle trainer; thus, slight pressure may have been applied to the expiration. Increasing respiratory muscle endurance was observed in respiratory muscle training, especially with hyperventilatory training [23, 24, 26], although we did not evaluate the respiratory muscle endurance.

### Effect of IMLET on normoxic exercise

With regard to exercise performance under N condition, numerous studies have observed that the exercise performance improved following the respiratory muscle training [20–22, 25, 32], while others disagreed [15, 24]. Since there was no significant difference in the extent of improvement in W_max_ between the IMLET group and the ET group, the improvement in W_max_ caused by the exercise training would not augment, even applying an inspiratory load during the training.

Hyperventilation helps maintain SaO_2_ during intensive exercise even under normoxia among the subjects with exercise-induced arterial hypoxaemia [33], implying that the gas exchange partly limits the *V*O_2peak_. Some studies reported that IMT improved exercise performance related to enhanced ventilatory response [17, 18]. Our result of the increasing *V*_Emax_ under normoxia following IMLET is consistent with those of previous studies [24, 27, 34]. However, this increase in *V*_Emax_ by IMLET did not contribute to the improvement of SaO_2_ and *V*O_2peak_, hence the exercise performance, because our subjects did not express arterial desaturation during maximal exercise. For instance, under N condition, *V*O_2peak_ did not change even if subjects were breathing helium–O_2_ mixtures with lowered air flow resistances [35].

### Effect of IMLET on hypoxic exercise

Esposito et al. [28] reported that *V*_Emax_ under H condition (11% O_2_) was increased following IMT compared with pre-training test, which is in agreement with our results. On the contrary, Downey et al. [15] reported that *V*_E_ under H condition (14% O_2_) during ~ 85% *V*_O2peak_ submaximal running was reduced following IMT. This disagreement of response in *V*_E_ following IMT might be due to a difference in test exercise intensity (maximal vs. submaximal). We observed that *V*_E_ was not significantly different between pre- and post-exercise tests when compared with the same absolute work load (at W_max_ in pre-test) (not shown in the results; 104.8 ± 22.0 L min^− 1^ in pre vs. 100.5 ± 26.6 L min^− 1^ in post; IMLET group). With this, we can speculate that an increase in *V*_Emax_ after IMLET was a result of an increase in work load after IMLET.

A high *V*_E_ under H condition appears to be beneficial for minimising the reduction in *V*O_2peak_ [4, 5], while WOB and oxygen cost of breathing should be higher with an increase in *V*_E_ [6] compared with those under N condition. Further, higher WOB would elicit respiratory muscle fatigue [11, 36]. Accordingly, we hypothesised that the benefit of IMLET would be emphasised under hypoxic exercise condition rather than under normoxic exercise condition. Previous studies have reported that the IMT did not change the *V*O_2peak_ under H condition [15, 28]. We observed an increase in *V*O_2peak_ in both training groups following the training period. Similar to the N condition, we cannot deny that the increase in *V*O_2peak_ under the H condition was caused by the exercise training itself. Interestingly, *V*O_2peak_ increases without any increase in SaO_2_ in both groups after training, which indicates that the magnitude of increase in *V*_E_ after IMLET is inadequate for improving the alveoli gas exchange. Thus, the increase in *V*O_2peak_ may be the result of exercise training causing circulatory function and peripheral adaptation. In fact, %d*V*O_2peak_ under H condition did not change even after IMLET. We should speculate one possibility that increase in *V*_Emax_ by IMLET was a result of an increase in exhaustion intensity by the training.

High *V*_E_ under H condition leads to higher O_2_ cost in respiratory muscles with an increase of inspiratory flow resistive work and expiratory flow limitation [36]. This would lead to a compromised blood flow to active muscles during heavy exercise [8, 9]. Recently, we have reported that in exhaustive incremental running, higher *V*_Emax_ and exercise performance without any change in *V*O_2peak_ under hypobaric normoxic condition (492 mmHg with 32.2% O_2_ gas inhalation) than under normobaric normoxic condition (760 mmHg) and estimated respiratory muscle *V*O_2_ reduced by 23% under hypobaric normoxic condition, suggesting that lower air density-related reduction of respiratory load affects exercise performance [37]. Further, Downey et al. [15] reported that during 85% *V*O_2peak_ running under H condition (14% O_2_), *V*O_2_ and cardiac output reduced after IMT. In line with this, we hypothesised that if IMLET reduced WOB against hyperventilation, the reduction in exercise performance under H condition would be attenuated. Our results that %dW_max_ was significantly smaller after training in the IMLET group partly supports the hypothesis that IMLET increased oxygen transport, such as increased blood flow to active muscles. Further, our result of an increase in *V*_Emax_ without any significant increase in WOB under H condition following IMLET, not seen in the ET group, implies that the participants can more hyperventilate with similar ventilatory effort after IMLET. Indeed, PEFR and VT at post-training test were higher than those at pre-training test, implying that breathing pattern is altered and increased with elastic work (i.e., recoiled energy work by chest wall inflation). However, we must emphasise the fact that WOB increased by 16% after IMLET. It is suspected that reduced %dW_max_ after IMLET is a result of reduced limb muscle fatigue due to unloading at submaximal intensity; eventually, the distribution of blood flow in the whole body *V*O_2_ at maximal intensity was not different before and after training. Further investigation is warranted to clarify this point.

### Limitations

This study has some limitations. Firstly, we did not fully record subject’s daily workout habits. However, subjects were instructed not to change their usual daily activities and not to participate in strenuous exercises. In addition, subjects belonged to the university track club, and similar exercise training was performed in both the IMLET group and ET group, except that in the laboratory. Secondly, subject’s fitness level and daily sports modality could have influenced our results. Further, the subjects with highest aerobic power tend to express the greatest decrease in *V*O_2pwak_ under H condition [5]. When dividing the subject’s groups, we ensured that the modalities of sports engaged are the same, and no significant difference was found in *V*O_2peak_ at the pre-training period between the training groups. Thirdly, exercise training was held only for 20 min per day. If the training duration is longer, the outcome between the ET groups and IMLET group may be different. However, the subjects were close to exhaustion by completing 20 min of exercise with the inspiratory load. Thus, subjects of the IMLET group may not be able to exercise further. Finally, the small number of subjects may have biased our conclusion. However, the minimum sample size was calculated from our study (power = 90%, α = 0.05). The minimum sample sizes were estimated to be 5, 9, 8, and 8 for PImax, *V*O_2peak_, *V*_Emax_, and W_max_ under H condition, respectively. Further, data collection needs to clarify the effect of IMLET on hypoxic exercise.

## Conclusions

Our results suggest that the exercise training with 50% PImax inspiratory load could improve inspiratory muscle strength and ventilatory response during hypoxic exercise. The extent of W_max_ decrease under H condition was smaller only in the IMLET group. This implies that IMLET can prevent an excessive decline in exercise performance under acute H conditions.

## Data Availability

Not applicable.

## Abbreviations

IMLET: Inspiratory muscle-loaded exercise training
IMT: Inspiratory muscle training
PEmax: Maximal expiratory mouth pressure
PEpeak: Peak expiratory pressure
P_ET_CO_2_: End-tidal partial pressure of CO_2_
SaO_2_: Arterial oxyhaemoglobin saturation
*V*_E_: Minute ventilation
*V*_Emax_: Maximal minute ventilation
*V*O_2_: Oxygen uptake
*V*O_2peak_: Peak oxygen uptake
VT: Tidal volume
WOB: Work of breathing
W_max_: Maximal work load
